# Structural transformation of a hydrogel-forming cell division protein ZapB of multidrug resistant *Klebsiella pneumoniae* with small molecules

**DOI:** 10.1371/journal.pone.0343254

**Published:** 2026-04-10

**Authors:** Ayesha Khan, M. Iqbal Choudhary, Sumaira Javaid, Humaira Zafar, Innokentiy Maslennikov, Atia-tul Wahab

**Affiliations:** 1 Dr. Panjwani Center for Molecular Medicine and Drug Research, International Center for Chemical and Biological Sciences, University of Karachi, Karachi, Pakistan; 2 H. E. J. Research Institute of Chemistry, International Center for Chemical and Biological Sciences, University of Karachi, Karachi, Pakistan; 3 Institute of Drug Discovery Technology, Ningbo University, Ningbo, Zhejiang, China; 4 Institute of Molecular Biology and Biochemistry, The University of Lahore, Lahore, Pakistan; 5 Chapman University School of Pharmacy, Harry and Diane Rinker Health Science Campus, Chapman University, Irvine, California, United States of America; NMIMS Deemed to be University - Mumbai Campus: NMIMS, INDIA

## Abstract

Septal ring assembly protein ZapB (EMR18431.1) is involved in Z-ring formation, and nucleoid segregation during the bacterial cell division . It promotes cell division in the initial stages of the cell cycle through direct interaction with FtsZ, thus stimulating Z-ring assembly. The ZapB inhibition can make bacterial cell susceptible to primary antibiotics, and therefore considered as an important approach for adjuvent therapy against MDR bacterial infections. Hence in the current study, ZapB protein of multidrug resistant *Klebsiella pneumoniae* was cloned, and expressed in *E. coli* system. To understand its role, and to assess the mechanism of ZapB activation, the characteristic microstructure of ZapB filament network were analyzed by scanning electron microscopy (SEM). ZapB self assembles into long filaments in a concentration dependent manner, and arranges themselves in meshworks, leading to polymeric forms. Furthermore, the effects of 26 compounds, including US-FDA approved drugs, and synthetic compounds of various chemical classes, on the filament formation ability of ZapB were studied by using SEM, and *in silico* methods. Among them, 5 compounds were found to disrupt the microstructure of ZapB, including trimethoprim (**1**), amikacin sulphate (**2**), hydroquinone (**3**), tobramycin (**4**), and *bis*(4-hydroxyphenyl) sulfide (**5**). Quantitative analysis demonstrated that morphological parameters of ZapB filament were significantly reduced following - treatment with test compounds. Among them, compound **2** showed the most pronounced effect, reducing filament length to 35.48 ± 21.55 µm (mean ± SD), whereas untreated ZapB filaments have an average length of 205.38 ± 89.34 µm, and broadest average diameter of 23.67 ± 7.71 µm. Molecular docking studies further predicted their non-covalent interactions with ZapB protein *via* hydrogen bond, salt bridges, and aromatic hydrogen bonds. Better understanding of the hydrogelation mechanism of ZapB, and its disruption is important for the development of new inhibitors of the cell division of bacteria. This can, therefore, serve as an important approach for the multi-target treatment of bacterial infections.

## Introduction

*Klebsiella pneumoniae* is one of the key nosocomial pathogens with MDR clones, containing extended spectrum β-lactamase enzymes (ESBLs) [1]. These enzymes are responsible of resistance against a broad range of β-lactam antibiotics [2]. Thus, only limited antibiotics are available against infections, caused by MDR *K. pneumoniae.* Therefore, new treatment strategies against MDR infection are urgently needed.

The common human pathogen *K. pneumoniae* is a Gram-negative intestinal bacterium that is closely related to *Eschericia coli* [3]. Bacterial cell division is a highly regulated process that requires highly synchronized actions of various proteins. The leading cytoskeletal protein, FtsZ, initiates the Z-ring formation with the help of various cell division and genome segregation proteins [4]. FtsZ protein of *K. pneumoniae* shares 98.7% sequence similarity with *E. coli* [3]. FtsZ assembles at the cytoplasmic membrane in a ring-shaped structure, known as the “Z ring.” Its attachment to the membrane is facilitated by two essential proteins, i.e., FtsA, and ZipA [5]. The intrinsic properties of FtsZ, in conjunction with the coordinated actions of multiple regulatory proteins, direct and concentrate the otherwise scattered FtsZ polymers at the site of nascent cell division [6]. Together, FtsZ, FtsA, and ZipA create the “proto-ring,” the first cytoplasmic stage in the *E. coli* divisome assembly [7]. The complex of FtsE, and FtsX proteins are also recruited at this point [8]. Additional divisome proteins are also recruited sequentially after a specified time delay, as presented below [9,10]:


FtsK → (FtsQ/FtsL/FtsB) → FtsW → FtsI → FtsN \]

Two of these proteins, FtsW and FtsI, are essential enzymes for the synthesis of septal peptidoglycans of dividing cells that are conserved in majority of walled bacteria. Both of these enzymes work as a pair, and thus referred as FtsWI [11]. A variety of accessory divisome proteins, including the Zap proteins [12], cell wall amidases, and their regulators, such as EnvC, are crucial for facilitating normal cell division [13].

The terminus region (Ter) macrodomain of bacterial chromosome contains a segment, known as *mat*S. During cell division, macrodomain Ter protein, MatP, that is vital for the organization and accurate segregation of the Ter macrodomain, binds to the *mat*S region. Consecutively, MatP interacts with FtsZ through a cell division regulatory protein, Z-ring- associated protein B (ZapB) [14]. The MatP-ZapB-FtsZ interaction is an important nexus that synchronizes the genome segregation, and bacterial cell division processes (S1 Fig in S1 File).

Misra and coworkers reported that duplication and regulation of bacterial cells, under biotic stress, is linked with the pathogenesis of bacteria; thus inhibiting the cellular function of proteins that promotes cell division, can be used to treat these infections [4]. Based on this hypothesis, we selected ZapB protein for inhibition, as it is involved in division and duplication of bacterial cells. ZapB is an abundant cell division factor involved in Z-ring formation, and nucleoid segregation with FtsZ. Its name originated from Z-ring- associated protein B [15]. There are approximately 13,000 copies of ZapB in each cell. It is engaged with the divisome through direct interaction with FtsZ, a crucial protein involved in the Z-ring assembly. Structurally, ZapB of *E. coli* (PDB ID: 2JEE) is a coiled-coil homodimer, made up of two alpha-helical polypeptide chains, with the ends binding the dimers with each other to form polymers [16,17].

ZapB plays an important role in the assembly of Z ring in *E. coli* (MG1655). Deletion of *zapB* gene slow down the cell division process, while increased expression of *zapB* results in the condensation of genetic material, followed by abnormal cell proliferation. *In vitro* studies reported that ZapB protein assembles in large filaments, and upon the addition of divalent cations (such as Mg^2+^, Ca) these filaments oligomerized into larger cables [16].

In the present study, we analyzed 13 US-FDA approved drugs, and 13 synthetic compounds of various chemical classes for the inhibition of ZapB. Briefly, ZapB from MDR *K. pneumoniae*-K6 (EMR18431.1) was cloned, expressed, and purified to investigate the effect of compounds on the protein microstructural properties using SEM, and *in silico* studies.

## Materials

All chemicals for cloning, expression and purification experiments were purchased from Biobasic (Canada), and ThermoScientific (USA). PCR machine of Bio-Rad (USA) was used for molecular experiments. Protein expression was performed on a New Brunswick *I*26 R (USA) incubator. Purification of the protein was achieved by using affinity column (HisTrap, GE Healthcare, UK) on AKTA purifier of GE Healthcare (UK). SEM analysis was performed by using Apreo 2 S LoVac instrument (Thermo Fisher Scientific, USA).

## Methodology

### Molecular cloning, and sequence validation

Genomic DNA of MDR *K. pneumoniae* (ATCC 700603) was purified by using genomic DNA extraction kit, following the manufacturer’s protocol (Thermo Scientific, K0512, USA). Based on the sequence of *zapB* gene, two primers, 5’- CCG GCC GGC ATG TCA TTA GAA GT-3’ (forward), and 5’-GGC TCT AGA TTA GAC TTC TTC CAT GC-3’ (reverse), were designed and synthesized (Biobasic, Canada). *zapB* Gene was amplified using pfu polymerase in 75 µL PCR reaction (S2 Fig in S1 File), and then extracted from gel using gene extraction kit (Biobasic, Canada). Purified gene and plasmid vector (carrying histidine tag) were subjected to double digestion by using restriction enzymes, *i.e.,* XbaI, and NgoMIV at 37 ºC. The temperature was then raised to 80 ºC for 20 min in order to deactivate the restriction endonucleases. Purification of digested gene/vector products were performed using clean-up kit (Qiagen, Germany). The *zapB* gene was then ligated into the vector using T_4_ DNA ligase enzyme at 24 ºC, resulting in the recombinant plasmid (construct). Transformation of construct in *E. coli* DH5α competent cells was performed, as described by the Sambrook and Russell [18]. Colony PCR of transformants was used to qualitatively confirm the integration of gene into the plasmid vector (S3 Fig in S1 File). The sequence of the cloned gene was verified through Sanger’s sequencing method from Macrogen (Korea) (S4 Fig in S1 File).

### Recombinant protein expression, and purification

The plasmid was further transformed, and expressed in *E. coli* BL21(DE3) strain. The protein expression was induced using 1 mM isopropyl β-D-1-thiogalactopyranoside (IPTG). Bacterial induced culture was then incubated in shaker incubator at 18 ºC for 18–20 hr with 180 rpm. After that bacterial cells were pelleted down at 3,900 rpm for 30 minutes at 4 ºC. Induced bacterial cells were lysed at 4 ºC using ultrasonic probe in lysis buffer (0.02 M sodium phosphate buffer, 0.2 M NaCl, 0.01 M imidazole, pH 7.5, and 1 mM phenylmethylsulfonyl fluoride (PMSF). The sonication pulse was burst for 6 sec with a delay of 12 sec at 50,000 Joules for 40–45 min. After sonication, cells were centrifuged at 14,000 × g at 4 ºC for 45 min, and supernatant was collected, filtered, and subject to purification. The SDS gel image of expression check of the protein is shown in S5 Fig in S1 File.

ZapB protein with His-tag was purified in a single step by affinity chromatography using HisTrap HP column (GE Healthcare, UK), and AKTA purifier FPLC system (GE Healthcare, Sweden) with equilibration buffer: 20 mM sodium phosphate, 200 mM NaCl, 10 mM imidazole, pH 7.5, and elution buffer: 20 mM sodium phosphate, 200 mM NaCl, and 500 mM imidazole, pH 7.5. The purified protein was eluted at 500 mM imidazole concentration, using one-step gradient. The protein was concentrated, and the buffer was exchanged to 20 mM sodium phosphate buffer (pH 6.7), 20 mM NaCl, through Amicon ultracentrifugal filters at 12 ºC using speed of 4,500 rpm (Millipore, Germany).

### Screening of test compounds

A total of 26 compounds, comprising of 13 US-FDA approved drugs, and 13 synthetic compounds of various chemical classes from the *in-house* PCMD Molecular Bank, were screened at 500 µM concentration, for their ability to inhibit the hydrogel formation of ZapB protein using SEM. *In silico* studies were also conducted to predict the intermolecular interactions between the ligands, and receptor (protein). List of compounds is provided in supplementary section (S1 Table in S1 File).

In current study, hydrogel spontaneously formed during concentrating the protein in 20 mM sodium phosphate buffer of pH 6.7 using Amicon ultracentrifugal filters at 12 ºC. The coiled coil architecture of ZapB is inherently prone to self-assembly into higher order structures, such as hydrogel *in vitro* [16].

### Analysis of microstructure of ZapB using Scanning Electron Microscopy (SEM)

10 µL of test samples were loaded on the silicon wafer (5 x 5 mm chips), and allowed to be adsorbed till dry at room temperature in a sterile environment. Conductive carbon tape was used to mount the dry substrate onto a SEM stub. A thin layer of gold conductive material was applied on the sample to enhance secondary electron emission for Scanning electron microscope (SEM) imaging. SEM (Apreo 2 S LoVac, Thermo Fisher Scientific, USA) was used to study the microstructure of ZapB protein alone, or with the addition of test compounds at 5 kV. The ratio of protein to test compounds was maintained 1:2. For the processing of image, the dwell time (scan speed) was set to 1–5 μs. To ensure comparability, a scale bar of 100 µm was fixed for all image analyses.

### Quantitative analysis of ZapB filaments

Quantitative analysis of protein filaments morphology was performed by measuring their length, diameter, and distribution in SEM images, using ImageJ (National Institute of Health, USA). Before analysis, images were calibrated using the embedded scale bar. Only well-resolved, non-overlapping filaments from more than one images were selected for the analysis. After compiling the measurements data for statistical analysis, and importing them into Origin software (OriginLab, USA), histograms were plotted to evaluate the distribution of filament diameter, and length in different conditions.

### Molecular docking

To predict the ligand-protein interactions at atomic level, molecular docking studies were performed. The crystal structure of ZapB (PDB ID: 2JEE) was prepared for the *in silico* studies using the Glide 6.9 module of the Schrödinger suite of programs, version 2023−2 [19,20]. The OPLS3 force field, and the protein preparation wizard tool were employed for protein preparation, and optimization. The LIGPREP tool in Maestro software was used to generate the protonation and potential tautomeric states of ligands [21]. The results were analyzed by the *Glide_XP* module to select the best docked pose. The docked poses were further validated by analyzing the binding free energies of the ligand-receptor complex using Molecular Mechanics/Generalized Born Surface Area (MM/GBSA) method [22].

## Results and discussion

**Fig 1:**
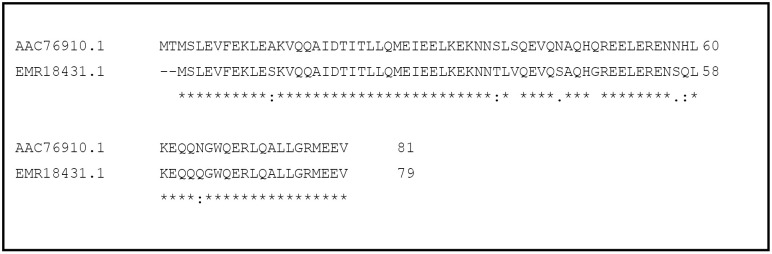
Sequence alignment of ZapB protein of non-pathogenic strain of *E. coli* with MDR *K. pneumoniae* using CLUSTAL OMEGA.

It was previously identified that in *E. coli,* the formation of Z-ring is stimulated by novel cell division factor ZapB [16]. Most of the available studies on ZapB are limited to its cellular localization, and qualitative visualization of polymer formation, while no research study was found that correlate drug-induced structural disruption of ZapB polymers with measurable changes in polymerization behavior. In the current study, ZapB protein of MDR *K. pneumoniae* was cloned, expressed, and purified for the investigation of its microstructure disruption using compounds of various chemical classes through SEM, and molecular docking. The sequence alignment of ZapB using Multiple Sequence alignment program of Clustal Omega from MDR *K. pneumoniae*-K6 (EMR18431.1) with non-pathogenic strain of *E. coli*-MG1655 (AAC76910.1) showed only minor differences at amino acids level (Fig 1)*.* The percent identity between the two sequences is 89.87%.

### Protein expression, and purification

**Fig 2:**
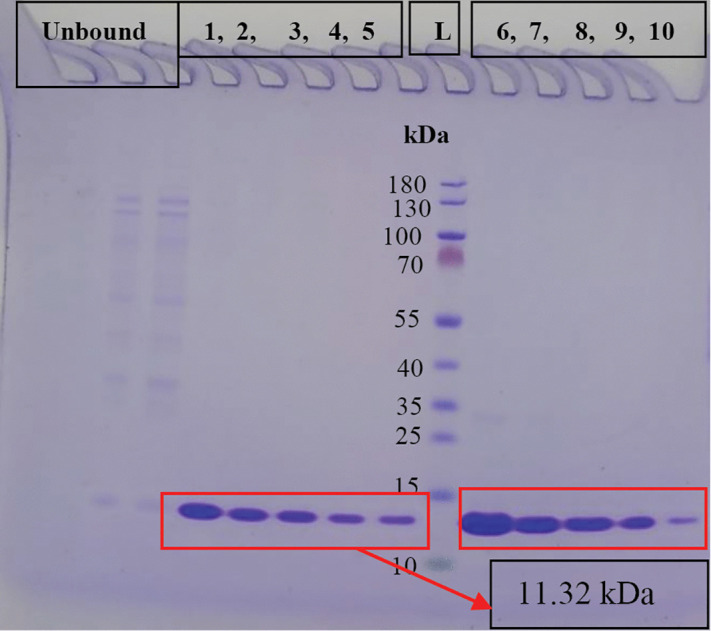
4-12% *Bis*-Tris SDS gel showing purified His-tagged ZapB protein (wells 1-10), eluted from HisTrap HP column at 100% B salt concentration, and L is protein ladder.

ZapB protein was found to be highly expressed. It was purified by using a single step purification strategy, *i.e.*, affinity chromatography with a final concentration of 5.18 mg/mL (Fig 2). It is known that hydrogel forming proteins cannot be obtained in good concentrations, in comparison to globular proteins, apparently due to their high viscosity in solutions [16]. Previous study reported that ZapB from *E. coli* cannot be easily concentrated more than 2 mg/mL [16]. In the current study, we successfully purified, and concentrated the ZapB protein to a higher concentration of 5.18 mg/mL.

### Microstructure of protein filaments

**Fig 3:**
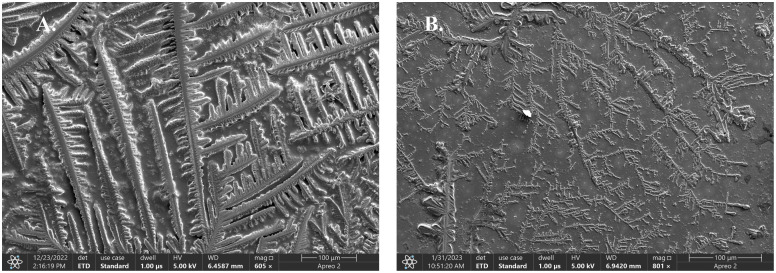
Scanning electron microscopic imaging of ZapB protein. (A) Concentrated form of 229 µM displaying long, and thick filaments. (B) Diluted form of 63 µM concentration showing short, and thin protein filaments.

Scanning electron microscopy (SEM) (Apreo 2) was used to analyze the formation of ZapB filaments, and their disassembly, after the addition of test compounds. According to SEM, ZapB protein exhibited an intense concentration-dependent oligomerization. Long, and thick filaments were observed in protein solution at 229 µM concentration (Fig 3A). Whereas, diluted ZapB protein at 63 µM formed short and thin filaments (Fig 3B). This study indicated that ZapB protein polymers are formed by its coiled-coil dimers, connected through their ends, thus appeared as branching structure on SEM images.

**Fig 4:**
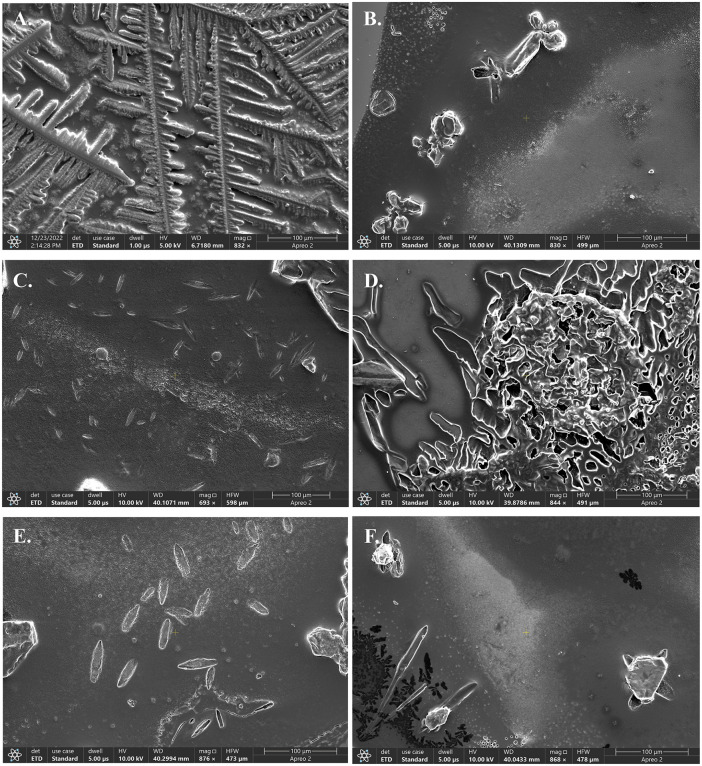
Scanning electron microscopic (SEM) images of ZapB protein (concentration 229µM) with various compounds at 500µ M concentration. (A) ZapB in native form showing protein filaments; ZapB incubated with (B) trimethoprim (1), (C) amikacin sulfate (2), (D) hydroquinone (3), (E) tobramycin (4), (F) *bis* (4-hydroxyphenyl) sulfide (5). All the images were captured at the same magnification scale.

The effect of US-FDA approved drugs, and synthetic compounds on ZapB filament disassembly was analyzed by SEM. Notably, 21 out of 26 compounds showed effect on ZapB filament microstructure (S6 Fig, and S7 Fig in S1 File), while 5 compounds, namely trimethoprim (**1**), amikacin sulphate (**2**), hydroquinone (**3**), tobramycin (**4**), and *bis*(4-hydroxyphenyl) sulfide (**5**), caused the disassembly of microstructures of ZapB at 229 µM protein concentration (Fig 4A to 4F). The disassembly of ZapB filaments, caused by drugs/ compounds, is reported here for the first time.

Trimethoprim (**1**) is an antibiotic used for the treatment of bladder infections, middle ear infections, and traveler’s diarrhea [23]. Trimethoprim works by blocking bacterial dihydrofolate reductase, and decreasing active tetrahydrofolates, which are crucial building blocks for the production of nucleic acids, and other biomolecules [24]. The current study identified that trimethoprim (**1**) was able to disrupt the long filaments of ZapB cell division protein, thereby indicating its potential role in the treatment of infections, caused by multidrug resistant bacteria Fig 4(B).

Amikacin sulfate (**2**) is one of the most commonly used aminoglycoside antibiotics. It inhibits the protein synthesis by blocking the initiation complex, needed to synthesize peptides, or by misreading of mRNA that ultimately synthesize non-functional proteins [25]. In this study, it was observed that protein filaments disassembled after treatment with amikacin sulfate (**2**) Fig 4 (C).

Hydroquinone (**3**) is a skin depigmentation agent. Clinically, it is used for the treatment of freckles, chloasma, melasma, solar lentigines, and post-inflammatory hyperpigmentation [26]. It’s mode of action is to inhibit melanin biosynthesis through impeding the conversion of L-3,4- dihydroxyphenylalanine to melanin by inhibiting tyrosinase enzyme [27]. In the current study, branched filaments of ZapB protein of *K. pneumoniae* were disrupted in the presence of hydroquinone (**3**) (Fig 4D).

Tobramycin (**4**) is a bactericidal drug of aminoglycoside class that binds irreversibly to the 30S bacterial ribosome to inhibit protein synthesis. Most Gram-negative bacteria are susceptible to compound **4** [28]. In current study, tobramycin (**4**) was able to disrupt the branched microstructure of ZapB protein, indicating its potential in the treatment of MDR related infections Fig 4E.

*Bis*(4-hydroxyphenyl) sulfide (**5**) consists of two 4-hydroxyphenyl groups, linked through a sulfur atom. It is used in pharmaceuticals industries as an antioxidant to enhance the stability, and effectiveness of active agents [29]. In current study, ZapB protein microstructure of MDR bacteria was disrupted by compound **5** (Fig 4F).

### Quantitative comparison of protein filaments

A clear description of ZapB filaments, and its disruption by test compounds was studied quantitatively by the analysis of SEM images. Results presented in Table 1 demonstrate that among 5 compounds, the most disruptive for the ZapB protein was compound **2**, as caused a major reduction in the length of protein filaments, *i.e.*, 35.476 ± 21.548 µm, as compared to the results obtained for protein only, *i.e.*, 205.38 ± 89.34 µm. Interestingly, the number of filaments for compound **2** was larger, but length was small. Then second most disruptive compound for the protein was compound **4** that caused fine filaments of protein with an average diameter of about 9.573 ± 3.383 µm. Compounds that did not disrupt the microstructure of ZapB, but showed mild effects on protein filaments were randomly selected for quantitative comparison.

**Table 1:** Morphological parameters of ZapB protein filaments.

Sample	Average Length ± SD (µm)	Average Diameter ± SD (µm)	N (Number of filaments measured)
ZapB protein only	205.38 ± 89.34	23.67 ± 7.71	29
**Compounds that disrupted the ZapB microstructure:** **(Protein 229 µm + compound 500 µm)**			
Protein + Compound **1**	55.855 ± 22.573	11.639 ± 6.298	20
Protein + Compound **2**	35.476 ± 21.548	9.995 ± 5.595	54
Protein + Compound **3**	67.832 ± 25.469	19.235 ± 5.901	24
Protein + Compound **4**	39.866 ± 26.924	9.573 ± 3.383	38
Protein + Compound **5**	66.033 ± 43.471	13.703 ± 3.618	21
**Randomly selected compounds (that did not disrupt the ZapB microstructure but showed mild effects) for quantitative analysis:** **(Protein 229 µm + compound 500 µm)**			
Protein + Compound **10**	158.456 ± 112.722	20.272 ± 4.779	38
Protein + Compound **11**	109.178 ± 78.783	13.209 ± 3.891	32
Protein + Compound **13**	176.222 ± 74.263	20.346 ± 5.001	33
Protein + Compound **22**	174.598 ± 98.991	21.477 ± 5.13	27
Protein + Compound **26**	132.026 ± 60.272	16.184 ± 4.517	34

The protein filaments distribution, according to their length and diameter, was evaluated by using histograms with mean values, and standard deviation (S8 Fig to S10 Fig in S1 File), as previously used in quantitative fiber imaging studies [30].

Quantitative interpretation of SEM images revealed measurable differences in protein filament length, and diameter across the analyzed conditions. Histogram analysis of filament length showed a shift toward shorter lengths in the compound-treated samples, as compared to the control group. Summary statistics indicated that the mean ± standard deviation of protein filament length differed significantly between various groups. Consistently, reduction in filament diameter was seen between the control, and compound-treated groups.

### Molecular docking of ligand-protein complex

To predict the interactions of selected compounds with ZapB at an atomic level, molecular docking studies were performed. The crystal structure of ZapB of *E. coli* (PDB ID: 2JEE) was selected as suitable template for homology modeling, because it showed 89.87% sequence similarity with the ZapB protein of MDR *K. pneumoniae*. All the drugs interacted *via* non-covalent interactions with the protein, including hydrogen bonds, aromatic hydrogen bond, and salt bridge formation. The docking scores, and binding energy are presented in Table 2. The negative values predicted through computational methods, indicating stronger binding affinity of the ligand with the protein.

**Table 2:** Representation of docking scores, and binding energies of compounds 1-5 against ZapB protein, predicted through *in silico* methods.

Compound	Docking score (kcal/mol)	MM/GBSA (kcal/mol)
**1**	−2.90	−26.76.
**2**	−0.16	−12.25
**3**	−5.11	−16.38
**4**	−3.78	−27.45
**5**	−3.79	−23.66

**Fig 5:**
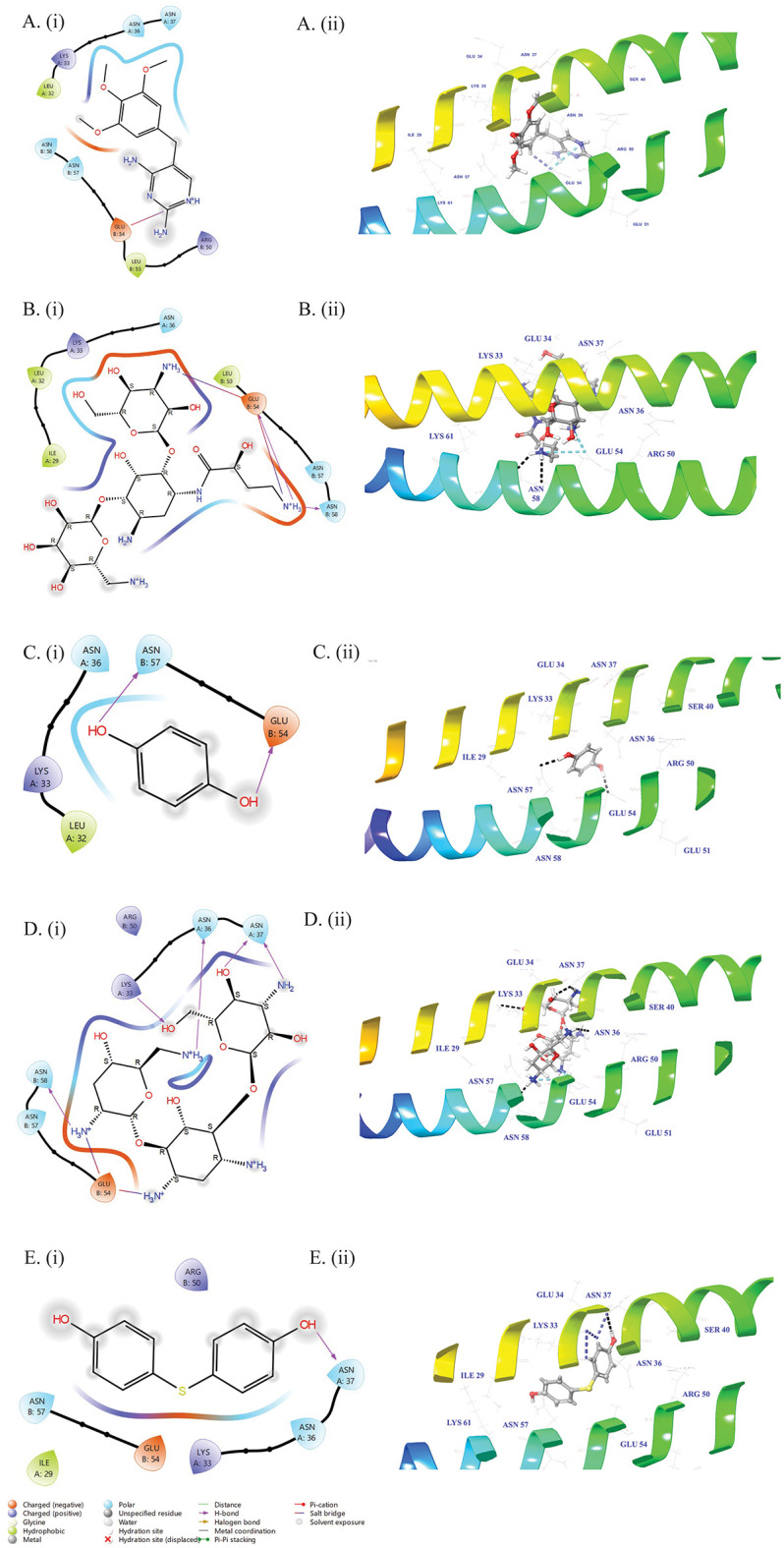
Docked poses of trimethoprim (1) (A. (i), A. (ii)), amikacin sulfate (2) (B. (i), B. (ii)), hydroquinone (3) (C. (i), C. (ii)), tobramycin (4) (D. (i), D. (ii)), and *bis* (4-hydroxyphenyl) sulfide (5) (E. (i), E. (ii)) with ZapB. (i) is the 2D Representation of ligand-protein profile and (ii) is the 3D representation of ligand-protein interactions in dotted lines.

Compound **1** showed interaction with Glu54 of protein through aromatic hydrogen bonding, involving 𝝅-H interaction between the aromatic ring of ligand, and the side chain of glutamic acid. It also formed a salt bridge through electrostatic interaction between its protonated amino group, and the negatively charged COO^-^ group of glutamic acid of protein (Fig 5 (Ai, Aii)).

Compound **2** showed a significant affinity for the binding site residues of target protein. It formed salt bridge with the carboxylate group of Glu54 bearing negative charge, indicating an ionic interaction is playing a major role in binding stability. Compound **2** also stabilized the ligand–protein complex by a hydrogen bond with the side chain amide group of Asn58 through polar interactions (Fig 5 (Bi, Bii)).

Compound **3** showed several hydrogen bonding interactions with the ZapB protein (Fig 5 (Ci, Cii)). It formed hydrogen bonds with the side chains of Asn57 of protein through polar interactions, and with Glu54 *via* electrostatic binding force. These interactions may contribute towards the stabilization of the ligand-protein complex, and suggest a strong affinity of compound **3** towards the binding site of protein.

Docking analysis revealed that compound **4** involved in several stabilizing interactions within the active site of the ZapB protein (Fig 5 (Di, Dii)). Compound **4** formed hydrogen bonds with the side chains of Asn36, Asn37, Asn58, and Lys33. These polar attractive forces appeared to be crucial for stabilizing the ligand within the binding site. Furthermore, a salt bridge was also predicted between compound **4,** and Glu54, involving ionic interactions between the ligand and the negatively charged carboxylate group of glutamic acid residue. The interactions suggest that compound **4** binds effectively with the target site of the protein, and suggest its potential as an inhibitor of the ZapB protein.

According to molecular docking studies, compound **5** forms several intermolecular interactions that enhanced its binding affinity with the ZapB protein (Fig 5 (Ei, Eii)). Asn37 participated in a hydrogen bond, and an aromatic hydrogen bond with the ligand, involving polar forces, and π-type interaction. Moreover, an aromatic hydrogen bond was also formed between the compound, and Lys33, indicating 𝝅-H interaction between the aromatic ring of ligand and the side chain of lysine. These interactions imply that *bis*(4-hydroxyphenyl) sulfide **(5)** interacted with ZapB in a favorable way, which contributes in the stabilization the ligand-protein complex.

Finally, it was predicted from the docking study that compound **4** showed the best binding free energy, closely followed by compounds **1,** and **5**, suggesting thermodynamically favorable and strong interactions with the target protein. While compound **3** with the lowest docking score, and moderate MM/GBSA value implied that the binding might be affected by enthalpic factors, and solvation effects. Compound **2** showed the least binding affinity, according to both docking and MM/GBSA scores. Therefore, compounds **1** (Trimethoprim), **4** (Tobramycin), and **5** (*Bis*(4-hydroxyphenyl) sulfide) predicted as strong disruptors of the target protein ZapB. The docking results for compound **4** are consistent with the *in vitro* findings, which showed that it caused the second most pronounced destabilization of the protein microstructure.

## Conclusion

Antibiotic resistance is a major global health challenge, and requires new strategies to treat infections by targeting novel biochemical pathways in pathogens. We demonstrated that thick and long filaments, formed by ZapB of *K. pneumoniae,* can be disassembled by 5 different compounds, including four US-FDA approved drugs, *i.e.*, trimethoprim, amikacin sulphate, hydroquinone, and tobramycin. The structural disruption of hydrogel-forming cell division protein ZapB, using selected compounds, was studied by SEM, and validated through quantitative analysis. Quantitative interpretation disclosed that amikacin sulphate (**2**) showed a strong disruptive effect, reducing protein filament length from 205.38 ± 89.34 µm (mean ± SD) to 35.48 ± 21.55 µm. Molecular docking studies revealed potential binding conformations of these compounds with ZapB, and also predicted the type of ligand-protein interactions, *i.e.*, non-covalent forces. This study reveals that compounds (trimethoprim, amikacin sulphate, hydroquinone, tobramycin, and *bis*(4-hydroxyphenyl) sulfide) can potentially inhibit bacterial cell division protein of MDR pathogens, and thus can serve as the leads for further studies as treatments against MDR *K. pneumoniae* infections.

## Supporting information

S1 File Supporting figures S1 to S10, S1 Table. (PDF)

S2 File Graphical abstract. (DOCX)
