# Supplementary material for: Structural transformation of a hydrogel-forming cell division protein ZapB of multidrug resistant Klebsiella pneumoniae with small molecules
Source: PLoS One. 2026 Apr 10;21(4):e0343254. doi: 10.1371/journal.pone.0343254 (PMC13068263; doi:10.1371/journal.pone.0343254)
Supplement: S1 File — (PDF) [file pone.0343254.s001.pdf]

Supporting Information:

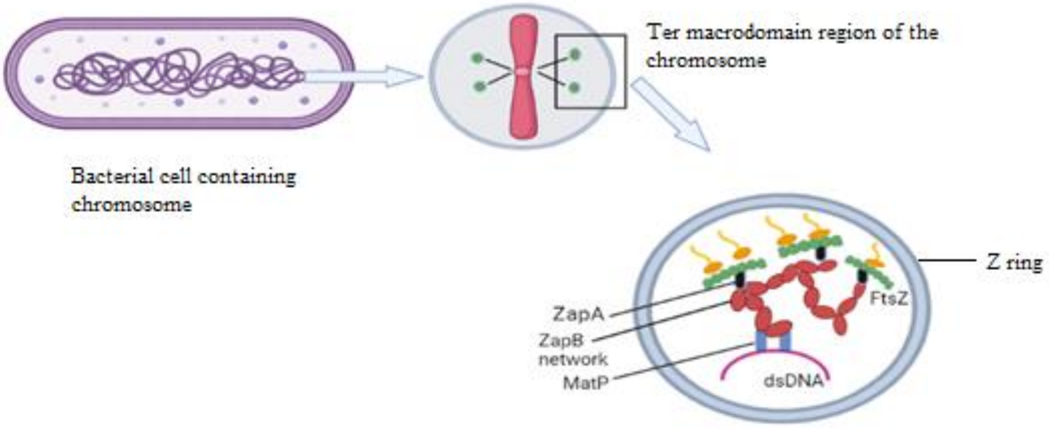

S1 Fig. Arrangement of the Ter linkage proteins in Z-ring.

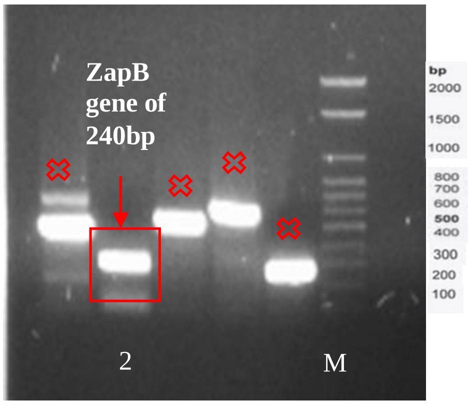

S2 Fig. Agarose gel analysis for PCR of targeted gene. Lane 2 represent the amplified gene product, while lane M is DNA ladder. (Irrelevant gene lanes are representing by cross sign).

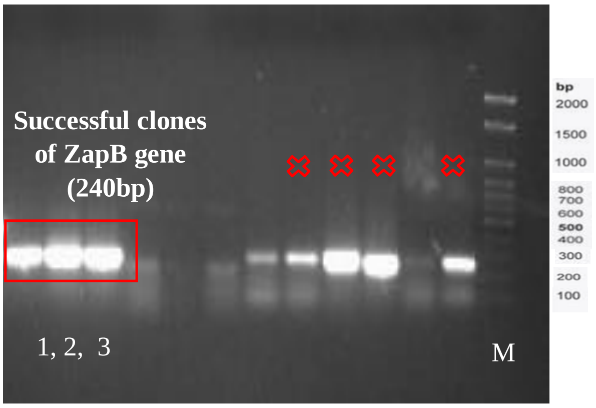

S3 Fig. Evaluation of colony PCR on agarose gel. Lanes 1, 2, and 3 present the amplified gene products, while lane M is DNA ladder. (Irrelevant gene lanes are representing by cross sign).

MSLEVFEKLESKVQQAIDTITLLQMEIEELKEKNNTLVQEVQSAQHGREELERENSQL
KEQQQGWQERLQALLGRMEEV

S4(i) Fig. Correct amino acid sequence of ZapB protein obtained on gene sequencing result.

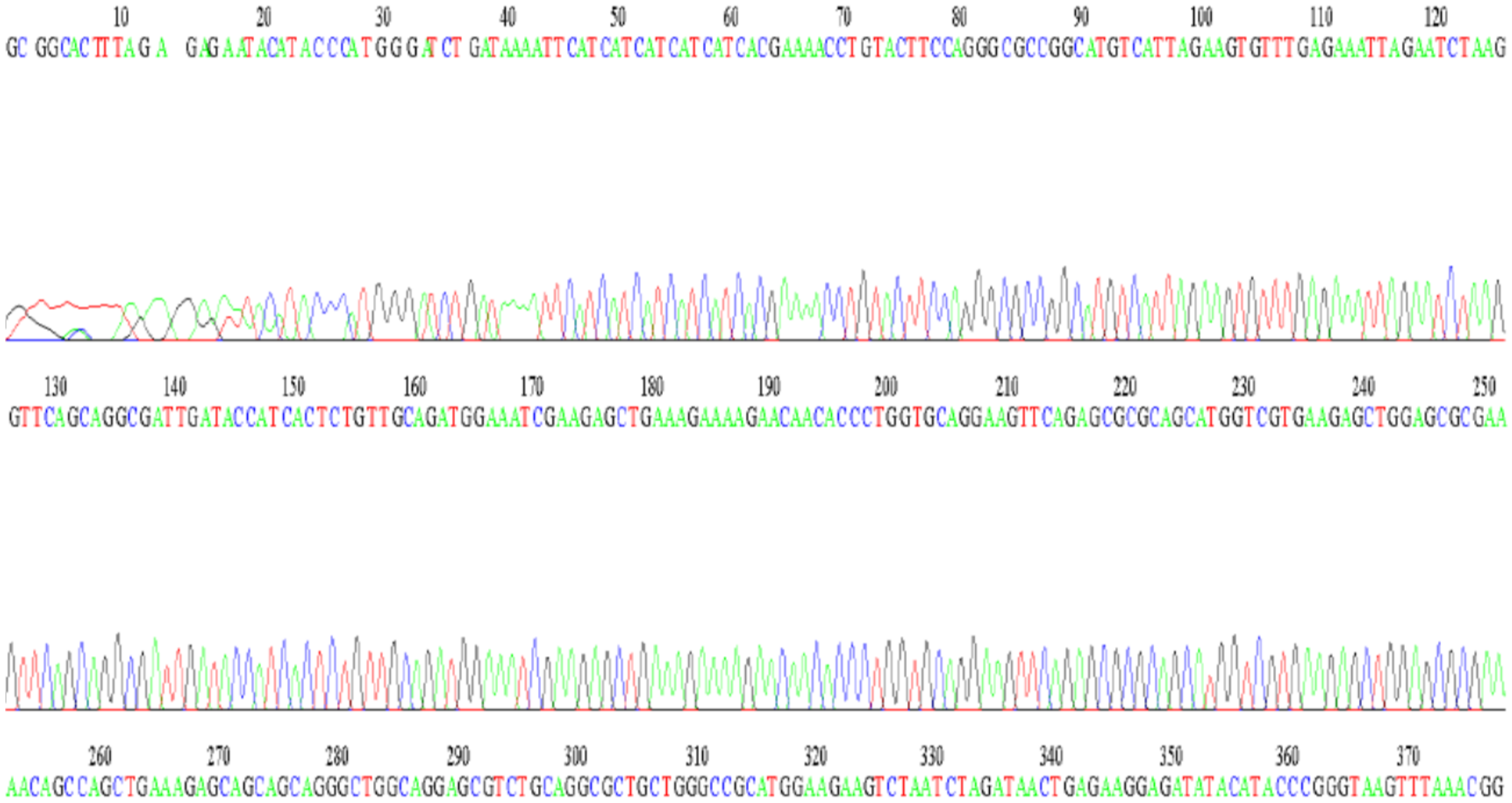

S4(ii) Fig. Electropherogram produced from Sanger´s sequencing of ZapB gene.

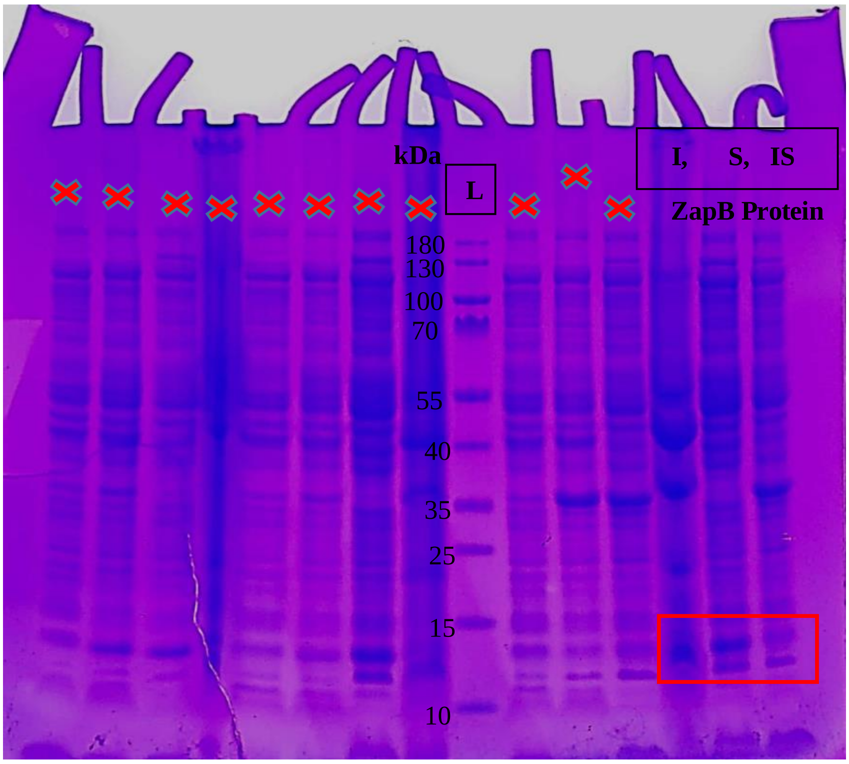

S5 Fig. SDS-PAGE analysis of protein expression. Lane I is induced, Lane S is soluble fraction, Lane IS is insoluble, and Lane L is molecular weight marker of protein in kDa. High expression of ZapB is evident from SDS gel.

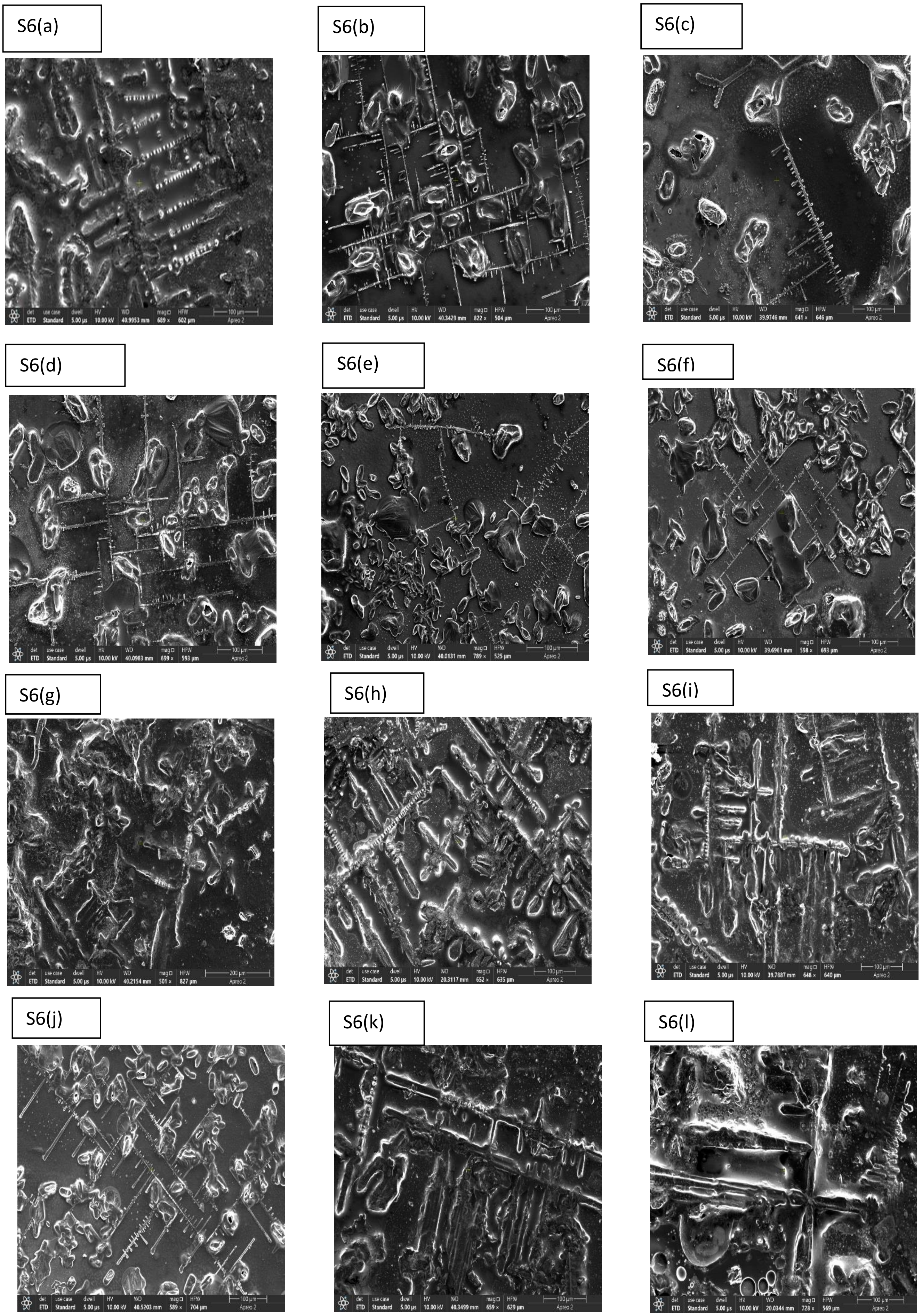

S6 Fig. Scanning electron microscopic images of ZapB protein with compounds of 500 μM concentration. ZapB was incubated with (a) Compound 11: 3-(4-hydroxyphenyl)-propionic acid , (b) Compound 18: 2,3,4-trihydroxybenzophenone, (c) Compound 17: 4'-amino-2',5'-diethoxy benzanilide, (d) Compound 19: 2-hydroxy-1,4-naphthoquinone, (e) Compound 20: Benzoin, (f) Compound 21: 4-methoxybenzoic acid, (g) Compound 12: 4(4-hydroxyphenyl)-2-butanone, (h) Compound 10: 2-(carboxymethyl) benzoic acid, (i) Compound 13: 4-ethylbenzoic acid, (j) Compound 16: 4-amino-2,6-dibromophenol, (k) Compound 8: 4-hydroxy-3-methoxybenzyl alcohol, (l) Compound 9: 2,4,6-trihydroxy benzoic acid that is displaying no inhibitory effect on protein microstructure.

S7(a)

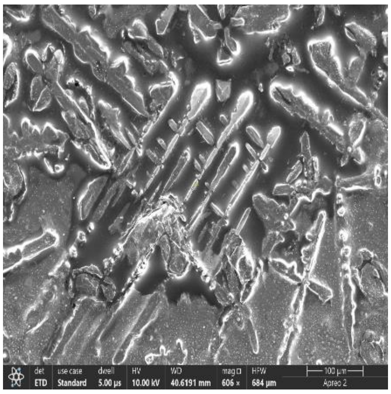

S7(b)

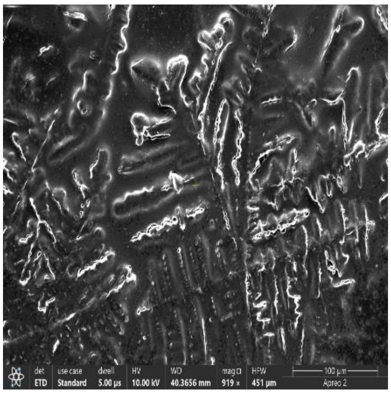

S7(c)

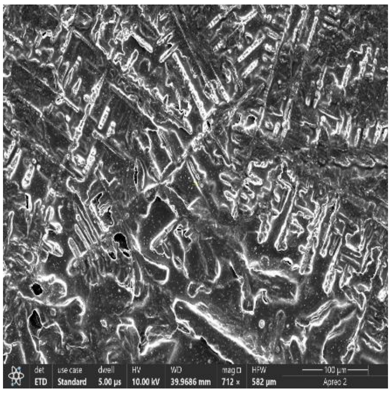

S7(d)

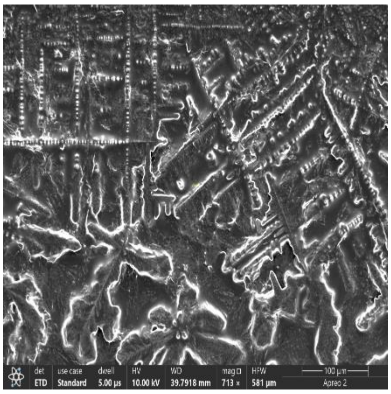

S7(e)

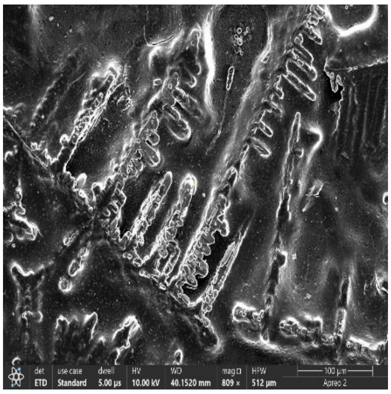

S7(f)

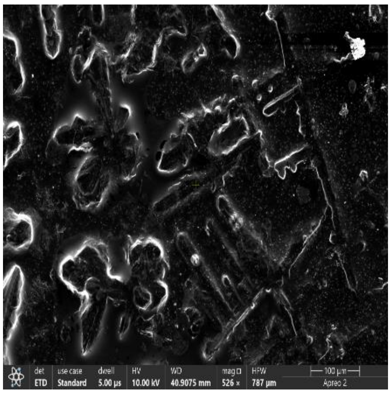

S7(g)

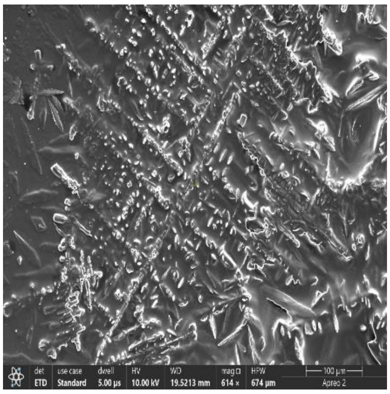

S7(h)

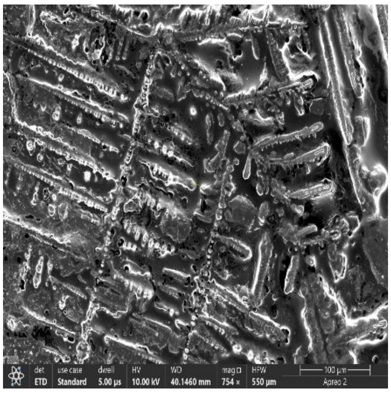

S7(i)

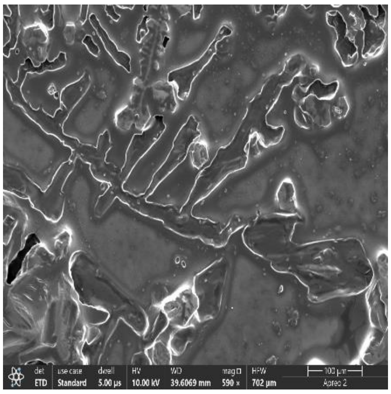

S7(j)

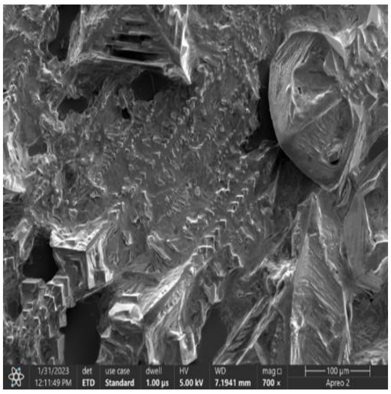

S7 Fig. Scanning electron microscopic images of ZapB protein with US-FDA approved drugs of 500 µM concentration. ZapB was incubated with (a) Compound 7: phenylephrine HCl, (b) Compound 26: citric Acid, (c) Compound 24: ascorbic acid, (d) Compound 23: ribavirin, (e) Compound 22: doxycycline, (f) Compound 15: isoniazid, (g) Compound 14: gentamicin Sulphate, (h) Compound 6: D-penicillamine, and (i) Compound 25: tetrahydrozoline HCl that is showing no inhibitory effect on protein filament formation. (j) Buffer of ZapB protein as blank.

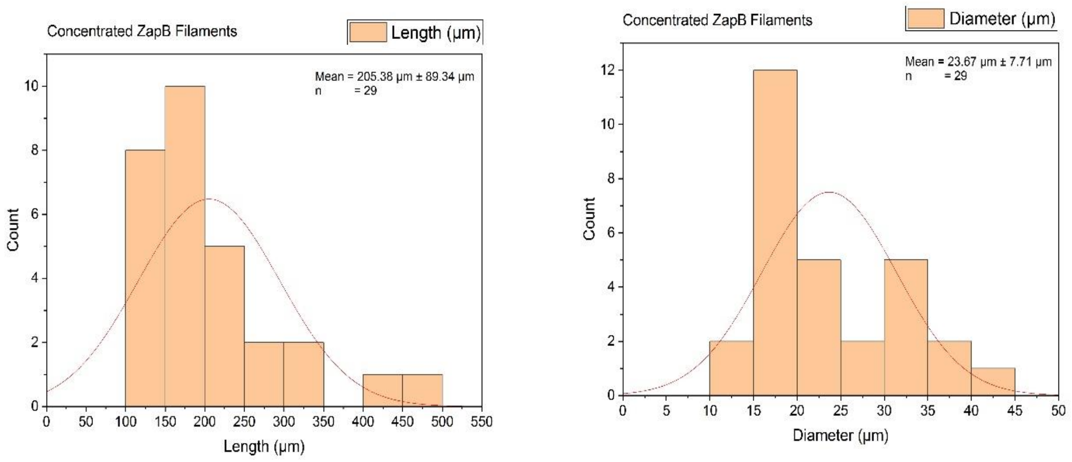

S8 Fig. Histogram analysis of concentrated protein filament length, and diameter as control.

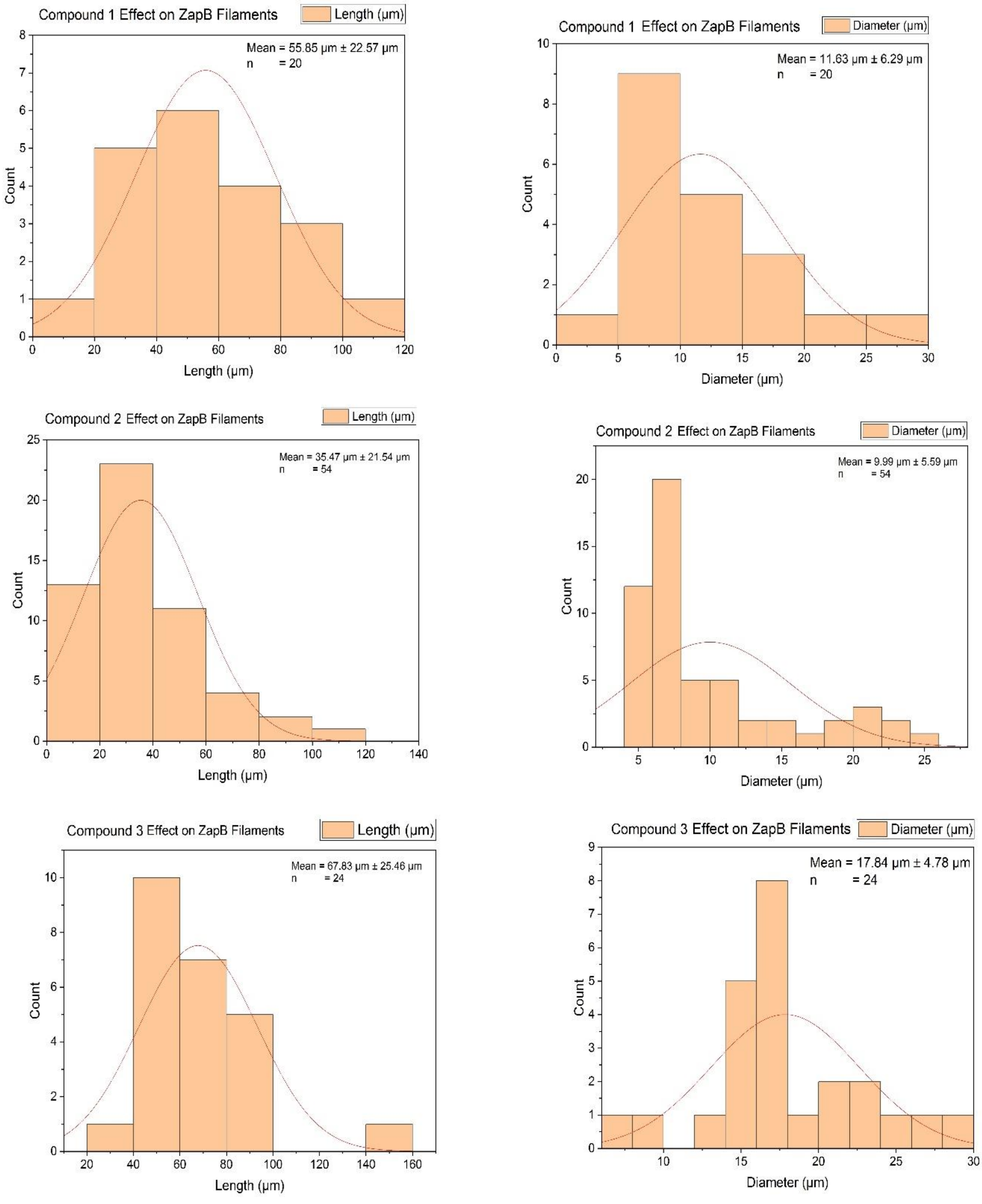
Compound 1 Effect on ZapB Filaments
Length (μm)
Mean = 55.85 μm ± 22.57 μm
n = 20
Count
Length (μm)
Compound 1 Effect on ZapB Filaments
Diameter (μm)
Mean = 11.63 μm ± 6.29 μm
n = 20
Count
Diameter (μm)
Compound 2 Effect on ZapB Filaments
Length (μm)
Mean = 35.47 μm ± 21.54 μm
n = 54
Count
Length (μm)
Compound 2 Effect on ZapB Filaments
Diameter (μm)
Mean = 9.99 μm ± 5.59 μm
n = 54
Count
Diameter (μm)
Compound 3 Effect on ZapB Filaments
Length (μm)
Mean = 67.83 μm ± 25.46 μm
n = 24
Count
Length (μm)
Compound 3 Effect on ZapB Filaments
Diameter (μm)
Mean = 17.84 μm ± 4.78 μm
n = 24
Count
Diameter (μm)

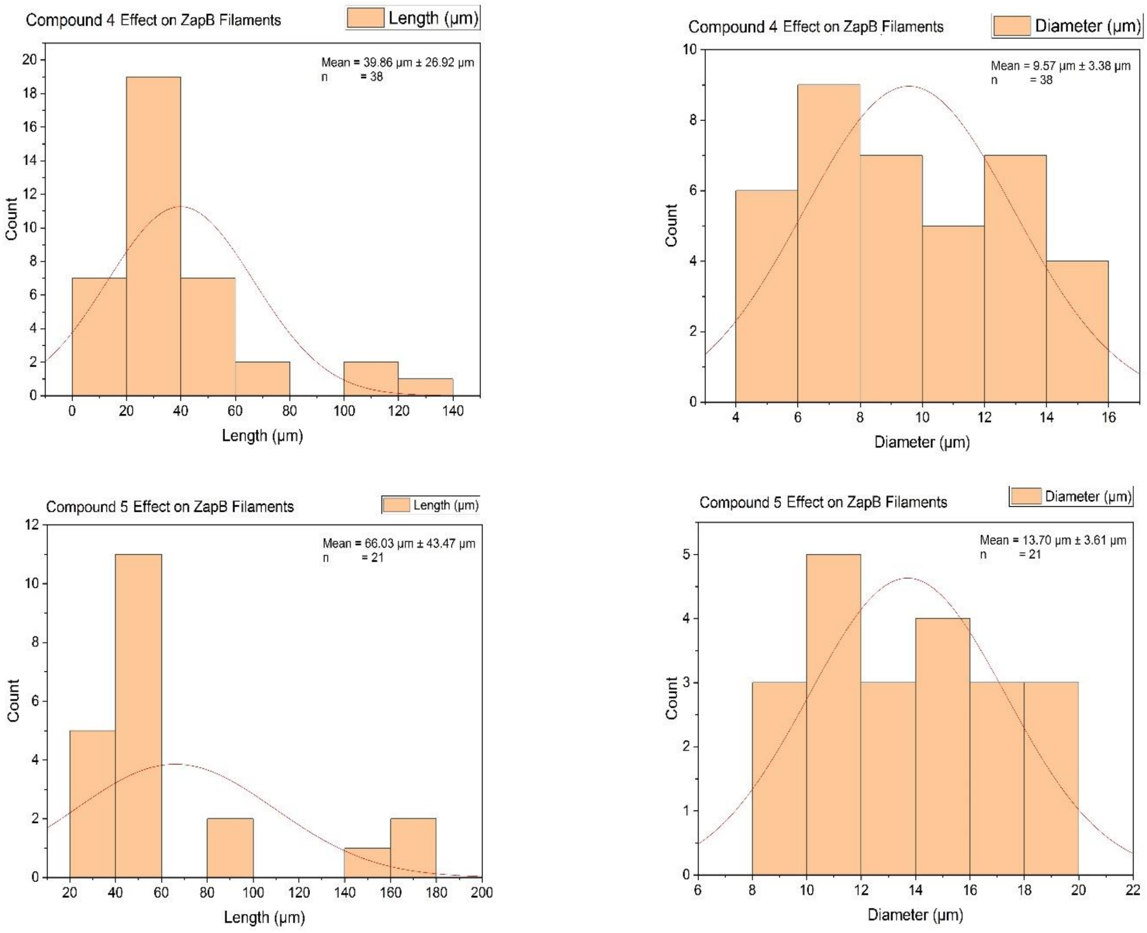

S9 Fig. Histogram analysis of protein filaments with compounds (1-5) that disrupted the microstructure of ZapB *in vitro*.

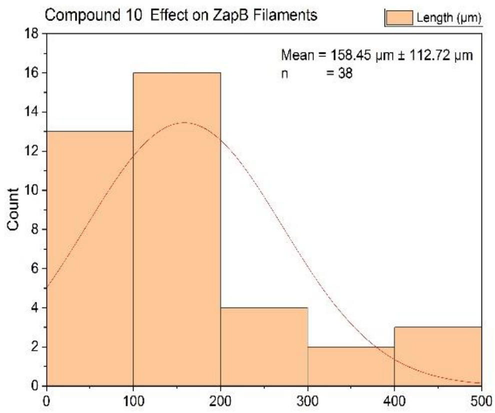
Compound 10 Effect on ZapB Filaments
Length (µm)
Mean = 158.45 µm ± 112.72 µm
n = 38
Count

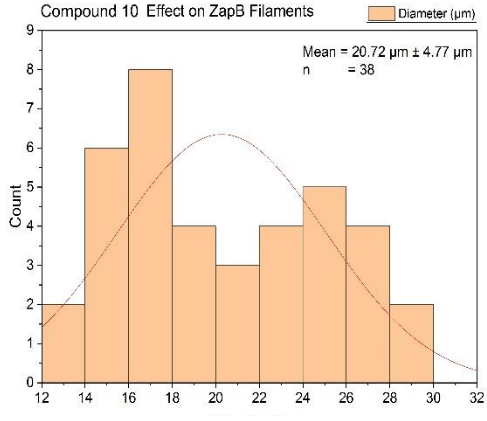
Compound 10 Effect on ZapB Filaments
Diameter (µm)
Mean = 20.72 µm ± 4.77 µm
n = 38
Count

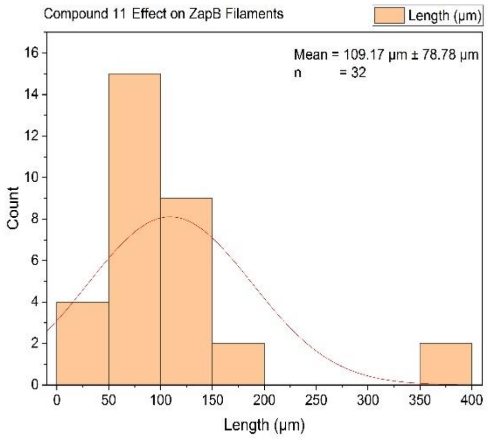
Compound 11 Effect on ZapB Filaments
Length (µm)
Mean = 109.17 µm ± 78.78 µm
n = 32
Count
Length (µm)

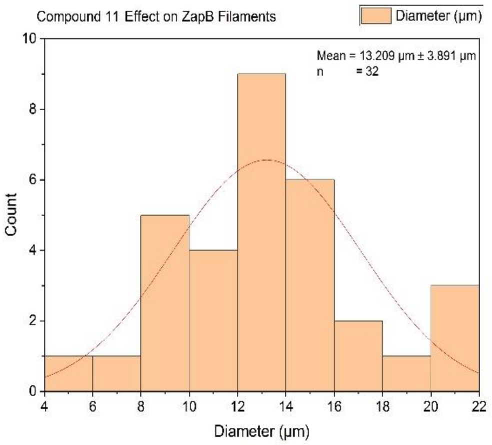
Compound 11 Effect on ZapB Filaments
Diameter (µm)
Mean = 13.209 µm ± 3.891 µm
n = 32
Count
Diameter (µm)

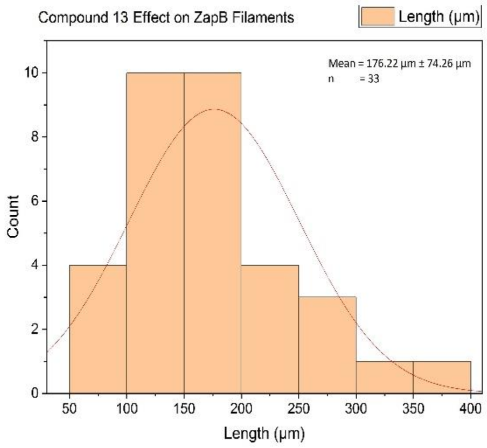
Compound 13 Effect on ZapB Filaments
Length (µm)
Mean = 176.22 µm ± 74.26 µm
n = 33
Count
Length (µm)

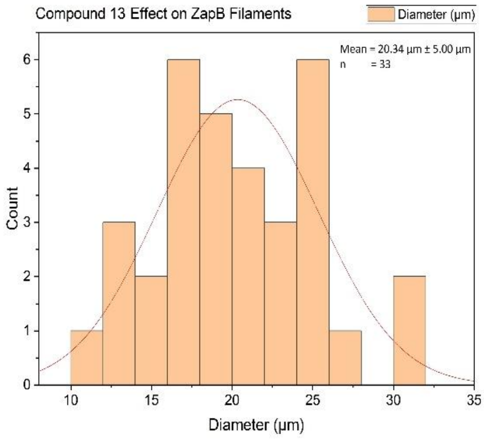
Compound 13 Effect on ZapB Filaments
Diameter (µm)
Mean = 20.34 µm ± 5.00 µm
n = 33
Count
Diameter (µm)

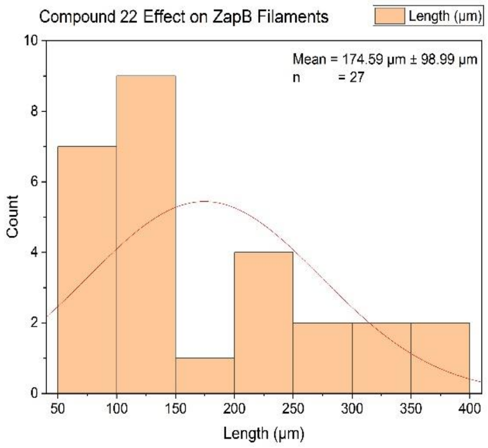
Compound 22 Effect on ZapB Filaments
Length (µm)
Mean = 174.59 µm ± 98.99 µm
n = 27
Count
Length (µm)

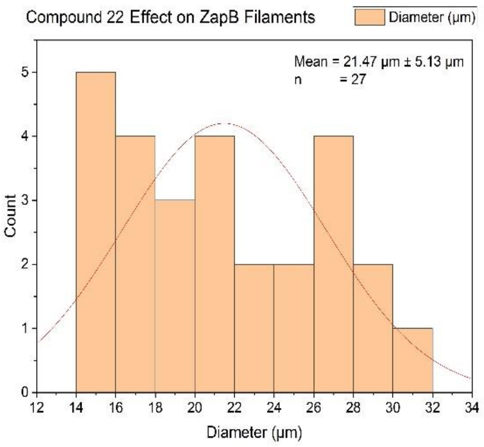
Compound 22 Effect on ZapB Filaments
Diameter (µm)
Mean = 21.47 µm ± 5.13 µm
n = 27
Count
Diameter (µm)

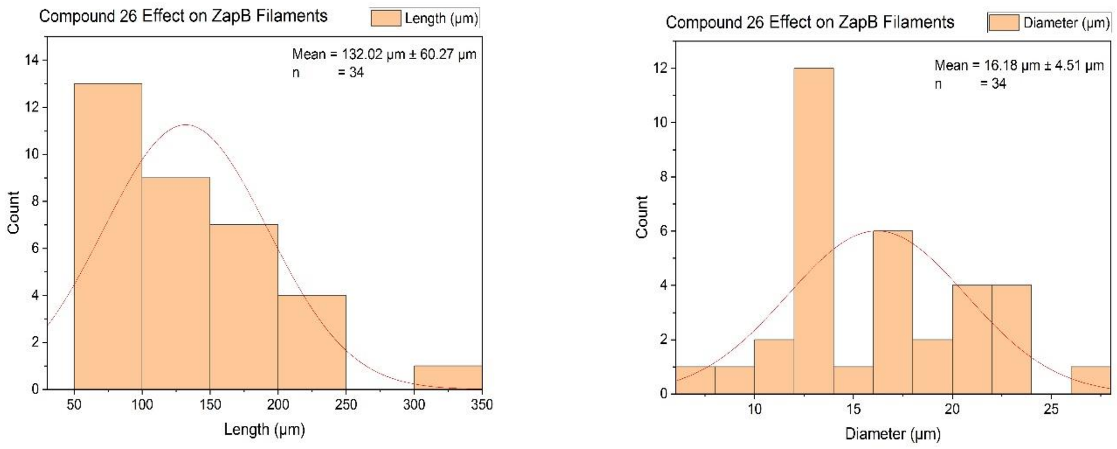

S10 Fig. Histogram analysis of protein filaments with compounds (10,11,13,22, and 26) that did not disrupt the microstructure of ZapB, but only showed mild effects *in vitro*.

S1 Table. Table of compounds screened against microstructure of ZapB protein.

Compounds	Name	Structure	Molecular weight	FDA/Non-FDA approved
**1**	Trimethoprim	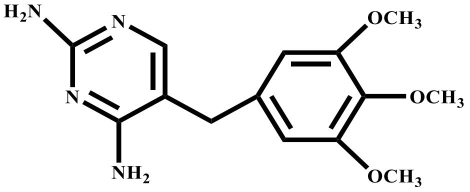	290.32	FDA approved
**2**	Amikacin sulphate	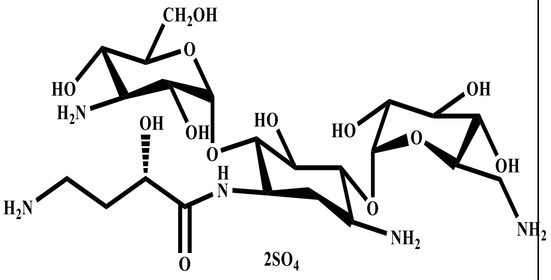	781.75	FDA approved
**3**	Hydroquinone	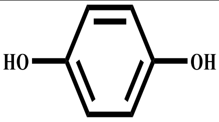	110.111	FDA approved
**4**	Tobramycin	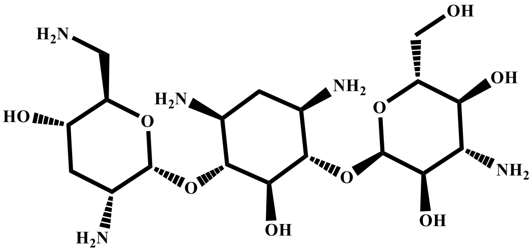	467.514	FDA approved
**5**	Bis(4-hydroxyphenyl)sulfide	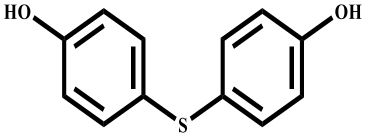	218.27	Non-FDA approved (synthetic compound)
**6**	D-Penicillamine	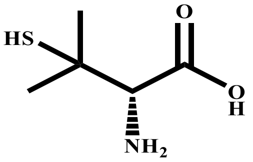	149.211	FDA approved
**7**	Phenylephrine HCl	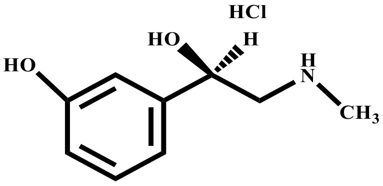	203.666	FDA approved
**8**	4-Hydroxy-3-methoxybenzyl alcohol	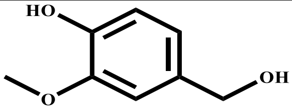	154.165	Non-FDA approved (synthetic compound)

**9**	2,4,6-Trihydroxy benzoic acid	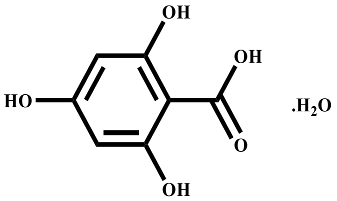	188.135	Non-FDA approved (synthetic compound)
**10**	2-(carboxymethyl) benzoic acid	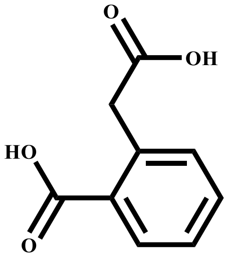	180.159	Non-FDA approved (synthetic compound)
**11**	3-(4-hydroxyphenyl)-propionic acid	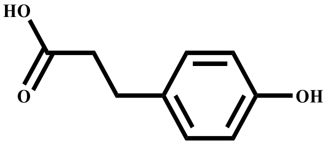	166.176	Non-FDA approved (synthetic compound)
**12**	4(4-hydroxyphenyl)-2-butanone	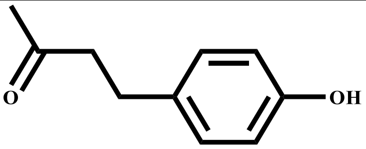	164.204	Non-FDA approved (synthetic compound)
**13**	4-Ethylbenzoic acid	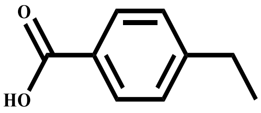	150.177	Non-FDA approved (synthetic compound)
**14**	Gentamycin Sulphate	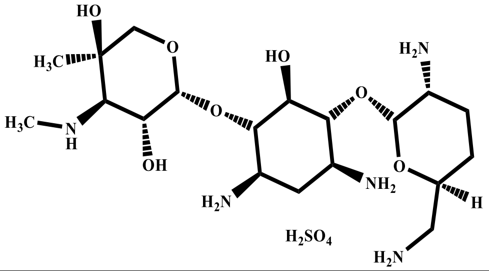	575.674	FDA approved
**15**	Isoniazid	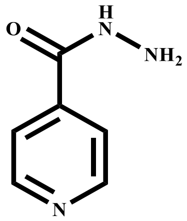	137.139	FDA approved
**16**	4-Amino-2,6-dibromophenol	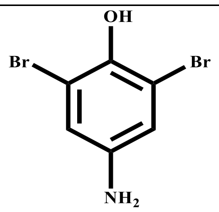	266.92	Non-FDA approved (synthetic compound)

**17**	4'-Amino-2',5'-diethoxy benzanilide	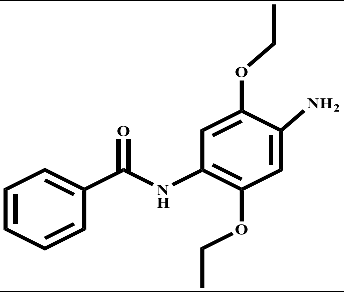	300.358	Non-FDA approved (synthetic compound)
**18**	2,3,4-Trihydroxybenzophenone	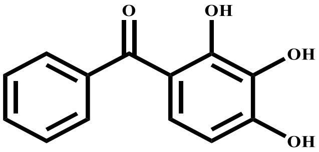	230.219	Non-FDA approved (synthetic compound)
**19**	2-Hydroxy-1,4-naphthoquinone	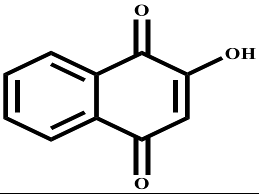	174.155	Non-FDA approved (synthetic compound)
**20**	Benzoin	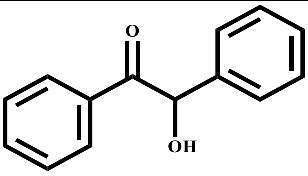	212.248	Non-FDA approved (synthetic compound)
**21**	4-Methoxybenzoic acid	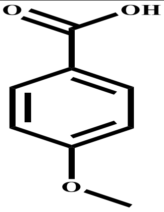	152.149	Non-FDA approved (synthetic compound)
**22**	Doxycycline	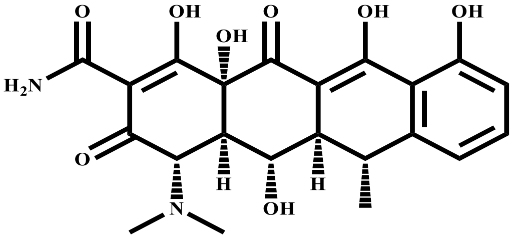	444.435	FDA approved
**23**	Ribavirin	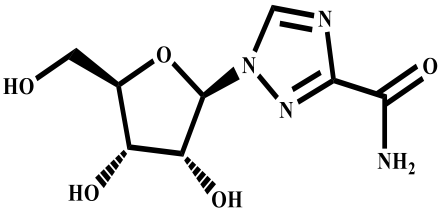	244.207	FDA approved
**24**	Ascorbic acid	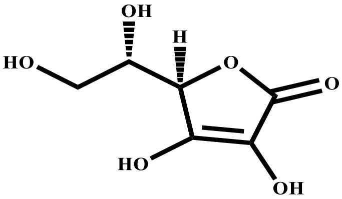	176.124	FDA approved

**25**	±Tetrahydrozoline HCl	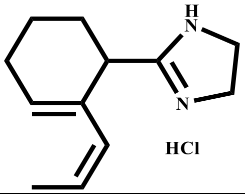	236.74	FDA approved
**26**	Citric Acid	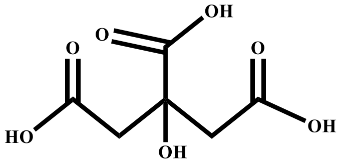	192.124	FDA approved
