# Supplementary material for: Structural transformation of a hydrogel-forming cell division protein ZapB of multidrug resistant Klebsiella pneumoniae with small molecules
Source: PLoS One. 2026 Apr 10;21(4):e0343254. doi: 10.1371/journal.pone.0343254 (PMC13068263; doi:10.1371/journal.pone.0343254)
Supplement: S2 File — (DOCX) [file pone.0343254.s002.docx]

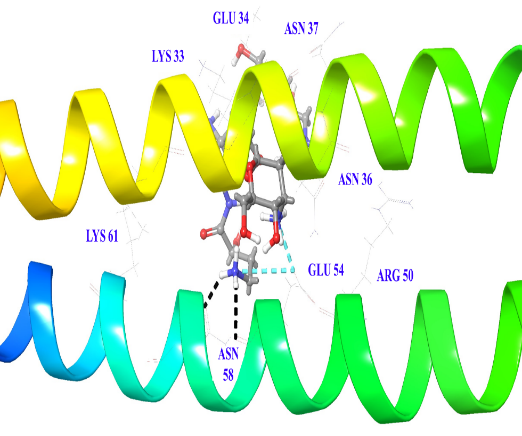

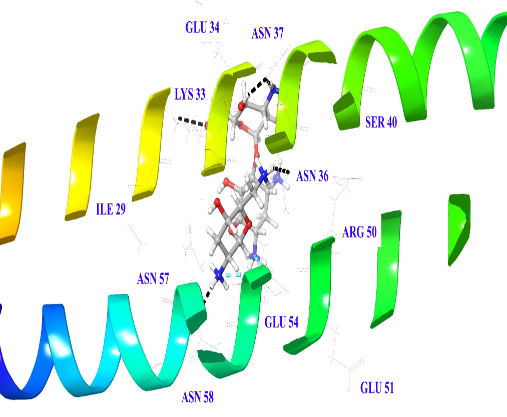

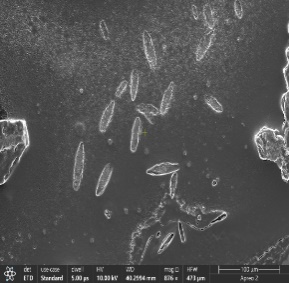

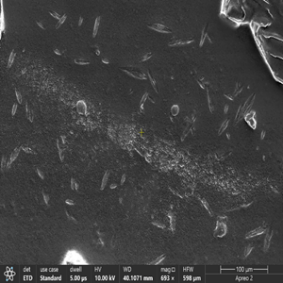


Protein only

Protein+compound **2**


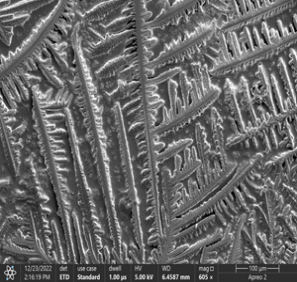

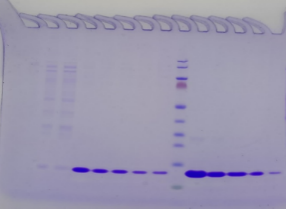

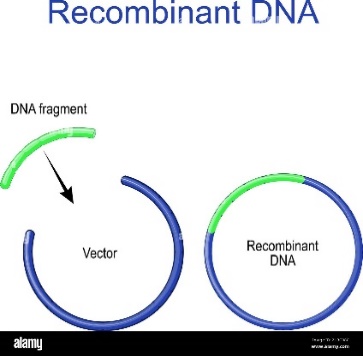


Microscopic observation of filament assembly and disassembly using SEM

Molecular docking studies showing interaction of drug with protein

**Septal ring assembly protein ZapB**

**of**

**MDR *K. pneumoniae***

Gene cloning, Protein expression, and Purification

Protein+compound **4**

Protein+compound **2**

Protein+compound **4**
